# Lung cancer classification and severity estimation using vision transformer-IPCNN with enhanced U-Net segmentation

**DOI:** 10.3389/frai.2026.1942753

**Published:** 2026-09-30

**Authors:** Nithya R, Vidhyapathi C M

**Affiliations:** School of Electronics Engineering, Vellore Institute of Technology, Vellore, Tamil Nadu, India

**Keywords:** Deep Learning (DL), Improved Parallel Convolutional Neural Network (IPCNN), Improved Wiener Filtering (IWF), Modified TransUNet (MTransUNet), Vision Transformer (ViT)

## Abstract

**Introduction:**

The death rate due to lung cancer is increasing because of late detection. Several automated lung cancer diagnostic techniques based on Machine Learning (ML) and Deep Learning (DL) have been developed for early detection. However, existing methods have limitations, including higher false-positive rates, lower sensitivity, and greater time consumption, which can affect early lung cancer diagnosis.

**Methods:**

To address these limitations, the Lung Cancer Classification and Severity Estimation–Improved Parallel Convolutional Neural Network-Vision Transformer (LCCSE-IPCNN-ViT) model is proposed. The framework consists of preprocessing, segmentation, classification, and severity estimation phases. First, the input images are preprocessed using the Improved Wiener Filtering (IWF) technique. The preprocessed images are then segmented using the Modified TransUNet (MTransUNet) model. Finally, the segmented images are classified and their severity is estimated using the IPCNN-ViT model, which combines the Improved Parallel Convolutional Neural Network (IPCNN) and Vision Transformer (ViT).

**Results:**

The proposed LCCSE-IPCNN-ViT model achieved an accuracy of 97.3%, sensitivity of 97.8%, specificity of 96.8%, precision of 96.8%, F-measure of 97.3%, and ROC-AUC of 98.5%.

**Discussion:**

The experimental results demonstrate that the proposed LCCSE-IPCNN-ViT model provides accurate and efficient lung cancer classification and severity estimation, indicating its potential for supporting early lung cancer diagnosis.

## 1 Introduction

Lung cancer is a disease that affects people more frequently. Globally, lung cancer cases as well as deaths have risen significantly (Nithya and Vidhyapathi, 2025). Of them, approximately 12%−15% of lung cancer cases are SCLC, and about 85–88% of lung cancer cases are SCLC (Burt et al., 2025). Early diagnosis and intervention in lung cancer are important to improve the 5-year survival rate because of the heterogeneity and invasiveness of lung cancer (Kumaran et al., 2024).

In recent years, diverse medical imaging techniques like Positron Emission Tomography (PET), chest X-ray, Computed Tomography (CT), Magnetic Resonance Imaging (MRI), Conventional Radiography (CRG), and Low-Dose Computed Tomography (LDCT) have been widely explored for lung nodule detection (Thakral et al., 2025). For lung nodule detection, CT is considered a gold standard imaging tool (Wang et al., 2024). However, it only identifies apparent lung cancer, with higher false-positive rates, along with producing harmful X-ray radiation (Mahmud et al., 2025). To reduce harmful radiation during lung cancer detection, LDCT was developed (Kang et al., 2025). However, cancer-related deaths are greatly concentrated in subjects who undergo LDCT (Bhatia et al., 2024). In addition, several new imaging techniques, especially MIT, have been developed for detecting cancer cells early. However, this approach lacks human-object clinical validation (Bortty et al., 2024).

Moreover, many CAD systems have been widely examined for the detection and categorization of lung cancer. When compared with trained radiologists, CAD systems offer the best cancer and lung nodule identification performance in medical images (Amin et al., 2024). In general, there are four steps in a CAD-based lung cancer detection approach, which are image processing, ROI extraction, feature selection, and classification (Noaman et al., 2024). From these steps, classification and feature selection have a crucial role in enhancing the CAD system's sensitivity and accuracy. However, it faces difficulty in classifying benign and malignant nodules (Musthafa et al., 2024).

Recently, many researchers employed Deep Learning (DL) techniques to assist radiologists in making accurate diagnosis (Noaman et al., 2024). Previous research has proved that DL-based CAD (Musthafa et al., 2024) systems can enhance the accuracy and efficiency of medical diagnosis, particularly for diverse common cancers diagnosis, including breast and lung cancers (Saha et al., 2024). From the original images, high-level features are automatically extracted using various network structures. However, these DL-based models also have some drawbacks, such as high false positives (FPs), low sensitivity, and higher time consumption (Liao et al., 2024). In recent years, methods have been developed that integrate image enhancement with Convolutional Neural Networks (CNNs) to improve cancer detection and diagnosis (Prasanth et al., 2024). To address the challenges of existing lung cancer detection methods, particularly their susceptibility to image noise, loss of fine-grained lesion details, inaccurate segmentation, high false-positive and false-negative rates, and limited sensitivity. These limitations can reduce the reliability of automated diagnosis and delay timely clinical intervention. Therefore, this study is motivated to develop an efficient and accurate framework that enhances CT image quality, accurately segments suspicious lung regions, extracts discriminative features, and effectively captures both local and global contextual information for reliable lung cancer classification and severity estimation. Consequently, we have introduced Lung Cancer Classification and Severity Estimation–Improved Parallel Convolutional Neural Network–Vision Transformer (LCCSE-IPCNN-ViT), which includes the following contributions:

- An Improved Wiener Filtering (IWF) technique is proposed for effective CT image pre-processing by reducing noise while preserving sharp boundaries and fine-grained structural details.
- A Modified TransUNet model is developed to provide accurate segmentation by incorporating residual connections, a Multi-Scale Depth-Wise Attention Gate (MSDAG) module, and Gaussian Error Linear Unit (GeLU) activation functions.
- An IPCNN model is proposed for discriminative feature extraction by integrating depth-wise separable convolution, Coordinate Attention (CA), and HardElish-Swish-Hyperbolic Tangent Function-Based Normalization (HE-S-HTFBN) modules, thereby enhancing feature representation while reducing redundant features.
- IPCNN-ViT develops a hybrid model to offer accurate classification and severity estimation by combining a parallel CNN and Vision Transformer.

This article is arranged as follows: Section 2 reviews several studies related to lung cancer detection or classification; Section 3 describes the LCCSE-IPCNN-ViT model; Section 4 presents this LCCSE-IPCNN-ViT model's experimental outcomes; and Section 5 provides the conclusion.

## 2 Literature survey

Some studies related to lung cancer detection and classification are reviewed below.

In 2025, Sankaranarayanan et al. (2025) proposed Lung Cancer Detection and Classification Using Two-Stream Conditional General Adversarial Network (LCDMIA-TCGAN), which was an enhanced lung cancer detection model. From the Lung Image Database Consortium and Image Database Resource Initiative (LIDC-IDRI) dataset, the input data was gathered. To remove noise from the CT images, pre-processing using Adaptive Tracking Dual Nested Kalman Filter (ATDNKF) was performed. Some key morphological features, such as perimeter, area, and eccentricity, were extracted using the Lifted Euler Characteristic Transform (LECT). For categorization of CT images, TCGAN was employed. By optimizing the TCGAN model's parameters using the Educational Competition Optimizer (ECO) algorithm, categorization accuracy improved.

In 2024, Mary and Thanammal (2024) proposed a lung cancer detection model named Deep Learning-based Lung Cancer Detection (DL-LCD). This model utilized two datasets, LIDC-IDRI and the National Institutes of Health (NIH) Chest X-ray dataset, which include Chest X-ray (CXR) and CT scan images. First, using Contrast Limited Adaptive Histogram Equalization (CLAHE), these images are pre-processed, leading to the elimination of noise and the enhancement of image clarity. Using the Honey Badger Algorithm (HBA), the lung images are segmented. Finally, these segmented images are categorized using DL. On the LIDC-IDRI database, this DL-LCD model attained 97.44% accuracy in abnormal lungs and 98.5% accuracy in normal lungs.

In 2024, Sangeetha et al. (2024) proposed a Multimodal Fusion Deep Neural Network (MFDNN) architecture for effectively integrating the data from diverse modalities (e.g., genomics, medical imaging, and clinical data), resulting in lung cancer diagnostic enhancement. Moreover, the accuracy and reliability of lung cancer diagnosis were enhanced by integrating Electronic Health Records (EHRs), clinical data, and multimodal approaches. This MFDNN attained an exceptional accuracy of 92.5%.

In 2025, Burhanettin Ozdemir et al. (2025) proposed a hybrid DL that incorporates CNNs with ViTs. Both large-scale and small-scale features in CT images are effectively captured by optimizing and incorporating grid and block Attention Mechanisms (AMs) with InceptionNeXt blocks. The utilization of InceptionNeXt blocks enables multi-scale feature processing, which makes the model more effective for complex and varied lung nodule patterns. During validation, an exceptional accuracy of 99.54% was achieved on the Iraq-Oncology Teaching Hospital/National Center for Cancer Diseases (IQ-OTH/NCCD) dataset.

In 2025, Shatnawi et al. (2025) proposed an Enhanced CNN for lung cancer prediction and classification using CT scans. Furthermore, this model utilized pre-trained models such as VGG16, ConvNeXtSmall, InceptionV3, ResNet50, and EfficientNetB0 to categorize lung cancer. This Enhanced CNN model attained 100% testing accuracy. This result highlighted the Enhanced CNN's potential in lung cancer detection.

In 2024, Crasta et al. (2024) proposed a DL framework for lung cancer detection and categorization in CT images. First, a three-dimensional (3D)-VNet architecture was proposed for accurate segmentation of pulmonary nodules. Afterward, a 3D-ResNet architecture was developed for their categorization. The Dice Similarity Coefficient (DSC) of 99.34% was achieved by the segmentation model, while reducing FP to 0.4% on the LUNA16 dataset. Moreover, the 3D-VNet network also provided better performance and robustness.

In 2025, Akintola et al. (2025) proposed Mask R-CNN for segmenting the lung nodules while CNN is utilized for malignancy classification. This study's main innovation was the development of a streamlined hybrid pipeline that automates both diagnosis and localization, reducing dependence on manual pre-processing. On a curated dataset, this model was trained, and for enhanced generalization, meticulous pre-processing and data augmentation techniques were applied. This model achieved precision and accuracy of 94.8 and 95.6%, respectively.

In 2023, Choudhury et al. (2023) introduced a Political Squirrel Search Optimization (PSSO)-based DL for the recognition and categorization of lung cancer. Here, Spine-Generative Adversarial Networks (GAN) was utilized for segmenting the lung lobe regions, and DNFN was utilized for forecasting the cancerous areas. Furthermore, Deep Residual Network (DRN) was used to determine cancer severity levels. This PSSO method was the combination of Political Optimizer (PO) and the Squirrel Search Algorithm (SSA) models. The dataset, which includes images from Lung Image Database Consortium (LIDC), was used for experimental validation.

In 2025, Ozdemir et al. (2025) suggested a hybrid Deep Learning model that uses both CNN and Vision Transformer architectures for detecting and classifying lung cancer in CT images. InceptionNeXt modules along with grid and block attention methods were used to capture the fine-grained multi-scale features of the images. It was trained on the Chest CT and IQ-OTH/NCCD datasets using transfer learning and image pre-processing approaches, achieving 98.41 and 99.54% accuracy, respectively.

In 2025, Sankaranarayanan et al. (2025) proposed the LCDMIA-TCGAN model for accurate lung cancer detection and classification from CT images. This model uses the LIDC-IDRI database, where the ATDNKF technique is used for noise reduction, and the LECT technique is used to extract morphological features, including area, perimeter, and eccentricity. TCGAN is used for classification into three classes of normal, benign, and malignant, and the ECO optimization technique is used for parameter tuning of TCGAN.

The advantages and challenges of several recent lung cancer detection or classification methods are presented in Table 1.

**Table 1:** Advantages, challenges, and results of a few recent lung cancer detection or classification methods.

Citation	Methods	Advantages	Challenges	Results obtained
Sankaranarayanan et al. (2025)	LCDMIA-TCGAN	Attained higher accuracy and computational efficiency with lower computational time	TCGAN's performance was sensitive to the diversity and quality of the training data, which affects its effective generalization ability.	Accuracy is 94%
Mary and Thanammal (2024)	DL-LCD	Attained 97.44 and 98.5% accuracy in abnormal and normal lungs, respectively.	Although it attained higher accuracy, severity levels are not mentioned.	Accuracy is 97.8%
Sangeetha et al. (2024)	MFDNN	Ensures the highest diagnostic accuracy along with minimizing the missed diagnoses.	Complex structure along with higher Computational time.	Accuracy is 92.5%
Ozdemir et al. (2025)	CNN + ViT	Provides a powerful and lightweight solution for early detection of lung cancer.	Higher model complexity along with higher computational cost.	Accuracy is 95.2%.
Shatnawi et al. (2025)	Enhanced CNN	It has the ability to accurately detect the case while minimizing errors.	Faces challenges like limited high-quality data along with technical constraints	Accuracy is 96%
Crasta et al. (2024)	3D-VNet	Lung nodules having diverse sizes and shapes are calibrated accurately with excellent robustness.	The model's dependability and precision still need to be enhanced.	Accuracy is 96.3%
Akintola et al. (2025)	CNN + Mask R-CNN	Helps radiologists in diagnosing lung cancer at an early stage.	Hybrid systems require considerable processing power along with resources.	Accuracy is 95.6%
Choudhury et al. (2023)	PSSO-based DL	Identifying lung cancer along with its severity level.	The model's performance can be affected by the local optima problem.	Accuracy is 89.3%
Ozdemir et al. (2025)	CNN-based ViT methods	The classification Accuracy is high	Computational complexity is high	Accuracy is 98.2%
Sankaranarayanan et al. (2025)	LCDMIA-TCGAN model	Reduced computational time	Limited generalization to different CT imaging conditions.	Accuracy improvement is in the range 30%−70%

### 2.1 Research gap

The late detection of lung cancer mainly causes cancer-related deaths. Recently, many methods have been developed for automatic detection and classification of lung cancer at an early stage. From the reviewed studies, several technical gaps are identified that motivate the development of the proposed LCCSE-IPCNN-ViT framework. Existing pre-processing methods may suppress high-frequency components and blur fine structural details, thereby affecting the detection of small and subtle lung nodules (Sankaranarayanan et al., 2025; Mary and Thanammal, 2024). Conventional segmentation approaches may also fail to preserve fine-grained local information while capturing global contextual features (Akintola et al., 2025). Several CNN-based methods extract redundant features and cannot represent long-range dependencies in complex CT images. The use of hybrid CNN-ViT models improves feature representation; however, they are associated with higher computational complexity (Sangeetha et al., 2024; Ozdemir et al., 2025). Moreover, the existing approaches are concerned with binary/multiclass lung cancer classification and do not provide accurate severity measurements. Another drawback is that some models are not able to generalize because of the reliance on a single image source or imaging condition. It should accurately segment lung lesions and extract both local and global features. These requirements motivated the development of the proposed LCCSE-IPCNN-ViT framework.

## 3 Proposed LCCSE-IPCNN-ViT model

Lung cancer has become a key global health concern in the last century, contributing to the majority of cancer diagnoses. Due to its rapid progression and higher mortality, early detection is crucial, as it can increase survival. However, current challenges like physician stress, poor image quality, ineffective communication, and inadequate information often affect early detection, which leads to higher medical costs and patient health deterioration. To tackle such challenges, an automatic lung cancer classification and severity estimation technique named LCCSE-IPCNN-ViT is introduced, which includes three phases: pre-processing, segmentation, classification, and severity estimation. Initially, to pre-process the input image, the IWF technique is proposed. Afterward, the MTransUNet model is proposed to provide accurate segmentation in the second phase. Finally, a hybrid model named IPCNN-ViT is proposed to provide effective lung cancer classification and severity estimation. The overall architecture of the LCCSE-IPCNN-ViT model is given in Figure 1.

**Figure 1:**
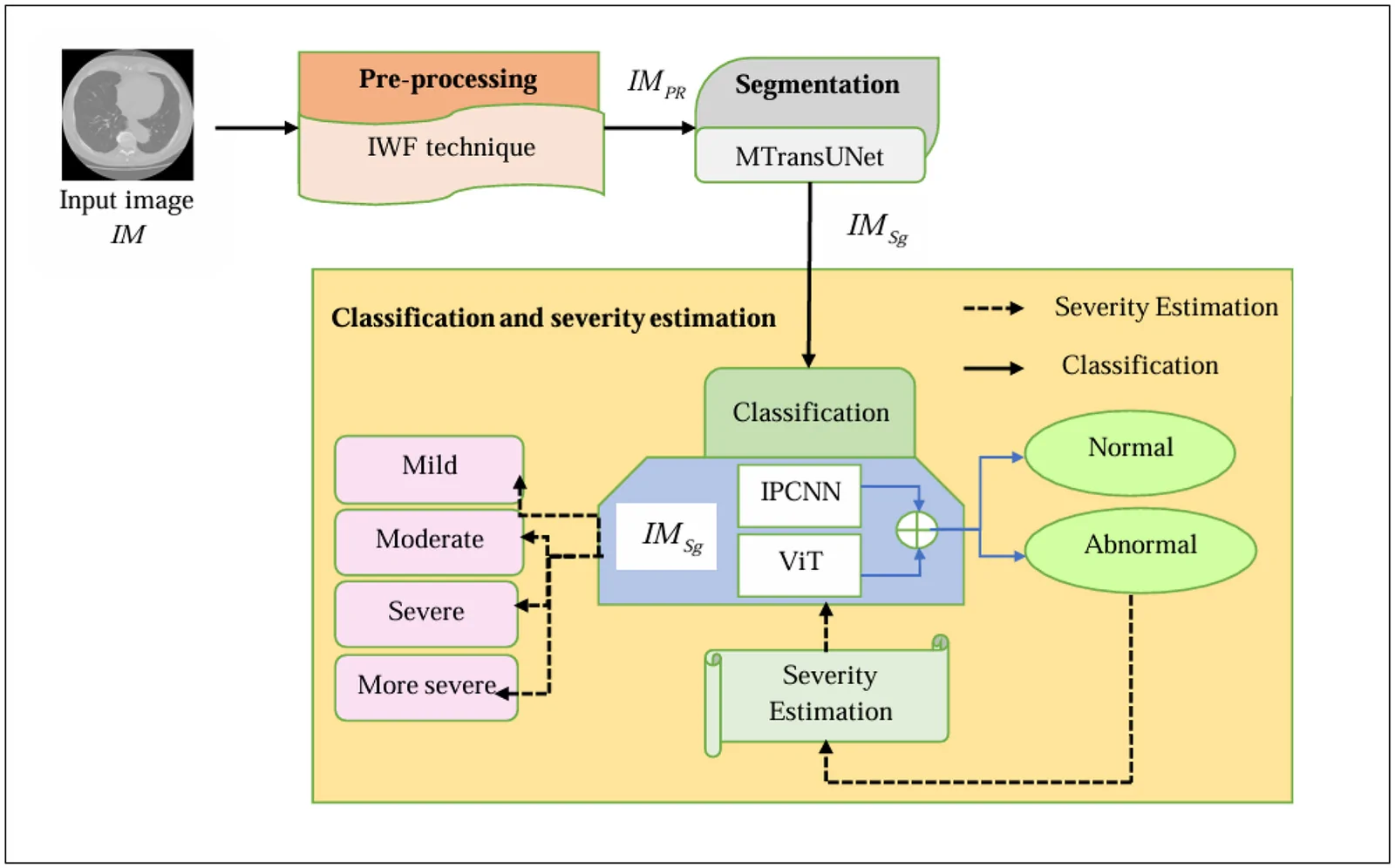
Overall architecture of LCCSE-IPCNN-ViT model.

### 3.1 Pre-processing

Image pre-processing is the initial phase, which includes applying operations to the input image*IM* for enhancing its quality and making it suitable for further analysis. Although many image pre-processing techniques, such as Gaussian, median, and average filters, are available, we have chosen the Wiener filter for our study due to its excellent noise reduction, good edge preservation, and adaptive behavior. A technique that is utilized to reduce the noise and blurring in images is known as Wiener filtering (Kalaivani and Phamila, 2018). However, these conventional Wiener filters smooth the high-frequency components of the image, blurring the edges and fine details. These blurred edges and fine details can reduce sharpness. To overcome this drawback, we have introduced an Improved Wiener Filtering (IWF) technique, which includes the following steps.

(i) Evaluate the power spectral density of the input image *IM* and the noisy image.
(ii) Apply a mask on a pixel of the noisy image.
(iii) Sort all the pixel's intensities that fall under the mask.
(iv) Calculate the median*Med*_(ν, *b*)_using Equation 1 and assign it to the mask's center pixel.
  $$\begin{array}{l} Med_{\left(\nu ,b\right)}=Median\left\{B_{\left(\nu ,b\right)}/\left(\nu ,b\right)\in W\right\} \end{array}$$ (1)
  where, *W* denotes the location (ν, *b*).
(v) Compute the Weighted Arithmetic Mean (*WAM*) as well as local variance $\left({\sigma^{2}}_{\underset{\left(\nu ,b\right)}{WAM}}\right)$ as in Equations 2, 3
  $$\begin{array}{lll} WAM & = & \frac{\underset{\nu b}{\sum }\left(B_{\left(\nu ,b\right)}.W_{\left(\nu ,b\right)}\right)}{\underset{\nu b}{\sum }\left(W_{\left(\nu ,b\right)}\right)} \end{array}$$ (2)
  $$\begin{array}{l} {\sigma^{2}}_{\underset{\left(\nu ,b\right)}{WAM}}=\frac{1}{\nu ,b}{\left(B_{\left(\nu ,b\right)}-WAM\right)}^{2} \end{array}$$ (3)
(vi) Evaluate the new pixel value *A*(*a, b*) using Equation 4:
  $$\begin{array}{l} A\left(a,b\right)=Med_{\left(\nu ,b\right)}+\delta_{\left(\nu ,b\right)}\times \left[\frac{{\sigma^{2}}_{\underset{\left(\nu ,b\right)}{WAM}}-V^{2}}{{\sigma^{2}}_{\underset{\left(\nu ,b\right)}{WAM}}}\right]\\ \;\times \left(B_{\left(\nu ,b\right)}-Med_{\left(\nu ,b\right)}\right) \end{array}$$ (4)
  where δ_(ν, *b*)_denotes the adaptive edge average weight, ${\sigma^{2}}_{\underset{\left(\nu ,b\right)}{WAM}}$denotes the local variance, while *V*^2^is the noise variance.
  In addition, δ_(ν, *b*)_can be computed as in Equation 5
  $$\begin{array}{l} \delta_{\left(\nu ,b\right)}=\frac{1}{1+e^{-\gamma \times {\sigma^{2}}_{\underset{\left(\nu ,b\right)}{WAM}}}} \end{array}$$ (5)
  where, γ is the smoothing parameter rating between (0,1).
(vii) Repeat the steps from (ii) to (vi) for all pixels of a noisy image.

Since this Improved Wiener Filtering technique replaces the pixel values of the mask with the median value, the variance changes based on WAM. The WAM gives higher weight to pixels near the center. Median value replacement is excellent for edge preservation, and using WAM instead of the mean, along with edge-aware weighting, reduces smoothing across edges, maintaining sharp boundaries and fine details. The resultant filtered image or pre-processed image is symbolized as *IM*_*PR*_.

### 3.2 . Segmentation using MTransUNet

In this phase, this *IM*_*PR*_ is subjected to the segmentation process. A computer vision technique that partitions the pre-processed image into numerous meaningful regions or segments by grouping pixels with similar characteristics. Although many segmentation methods are available, in the case of medical images, TransUNet can be a better solution because it is designed particularly for medical image segmentation TransUNet (Chen et al., 2021). TransUNet merges the transformers with the U-Net architecture. To capture the global context (long-range dependencies), this conventional TransUNet uses a transformer, and to recover spatial details, it utilizes a CNN-based decoder. However, while transformers capture global context well, they may lose fine local details, which can result in the loss of some fine-grained local spatial details critical for identifying small lung modules and subtle cancerous regions, thereby decreasing segmentation accuracy on small or complex structures. To tackle this challenge, an MTransUNet is proposed in this study, which is an improved version of the conventional TransUNet model. The architecture of MTransUNet-based segmentation is shown in Figure 2.

**Figure 2:**
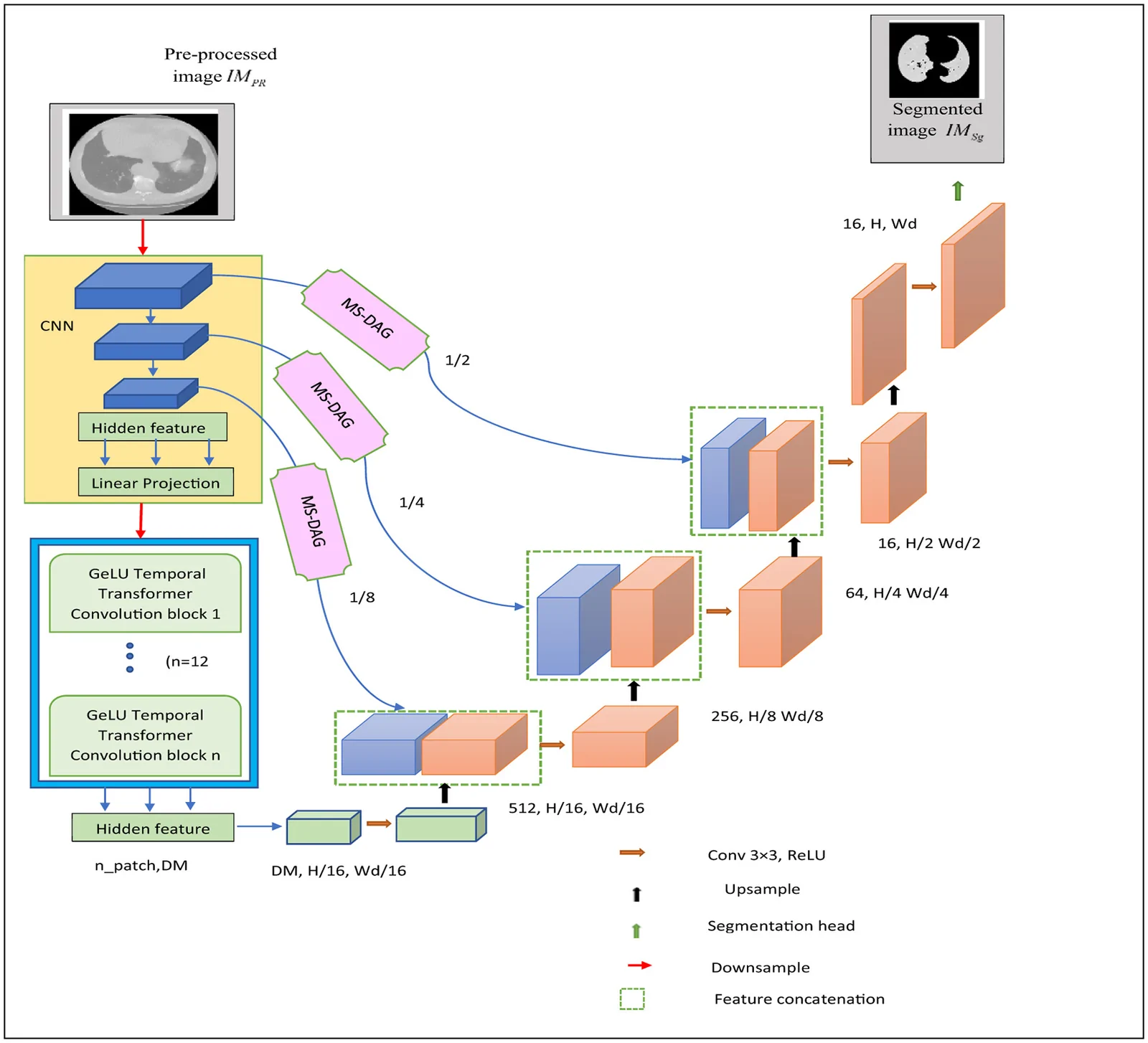
Architecture of MTransUNet-based segmentation.

Initially, *IM*_*PR*_ is given as the input to the MTransUNet model. An image $IM_{PR}\in RG^{H\times Wd\times Ch}$ has the spatial resolution of *H*×*Wd* and several channels *Ch*. The main goal is to predict the respective pixel-wise label map having the size *H*×*Wd*. For that, we have directly trained a CNN to encode the images into higher level feature representations. Afterward, they are decoded back to full spatial resolution. This MTransUNet structure includes three layers: a CNN-based encoder, a Transformer encoder, and a decoder.

#### 3.2.1 CNN-based encoder

First, *IM*_*PR*_is passed through some convolutional layers. These layers extract local spatial features. Afterward, the feature maps are divided into non-overlapping patches. Afterward, each patch is flattened and projected into a vector embedding. These vectorized patches *X*_*pt*_ are mapped into a latent *DM*-dimensional embedding space with the utilization of a trainable linear projection (Chen et al., 2021). For encoding the patch information, particular position embeddings that were added to the patch embeddings are learned to retain positional information as given in Equation 6,


$$\begin{array}{l} C_{0}=\left[X_{pt}^{1}E;X_{pt}^{2}E;,,,;X_{pt}^{2}E\right]+E_{Pos}, \end{array}$$ (6)

where patch embedding projection is *E*∈*RG*^(*P*^^2^.*CS*) × *DM* and position embedding can be denoted as $E_{Pos}\in RG^{N\times DM}$.

#### 3.2.2 Transformer encoder

The embedded patches are given as input to the transformer encoder. This layer is utilized to model the long-range dependencies. This transformer encoder layer includes *n* count of GeLU Temporal transformer convolutional blocks (GeLU-TTCBs). Each block has an MSA, layer normalization, GeLU-TCM, and feed-forward network layers, as shown in Figure 3.

**Figure 3:**
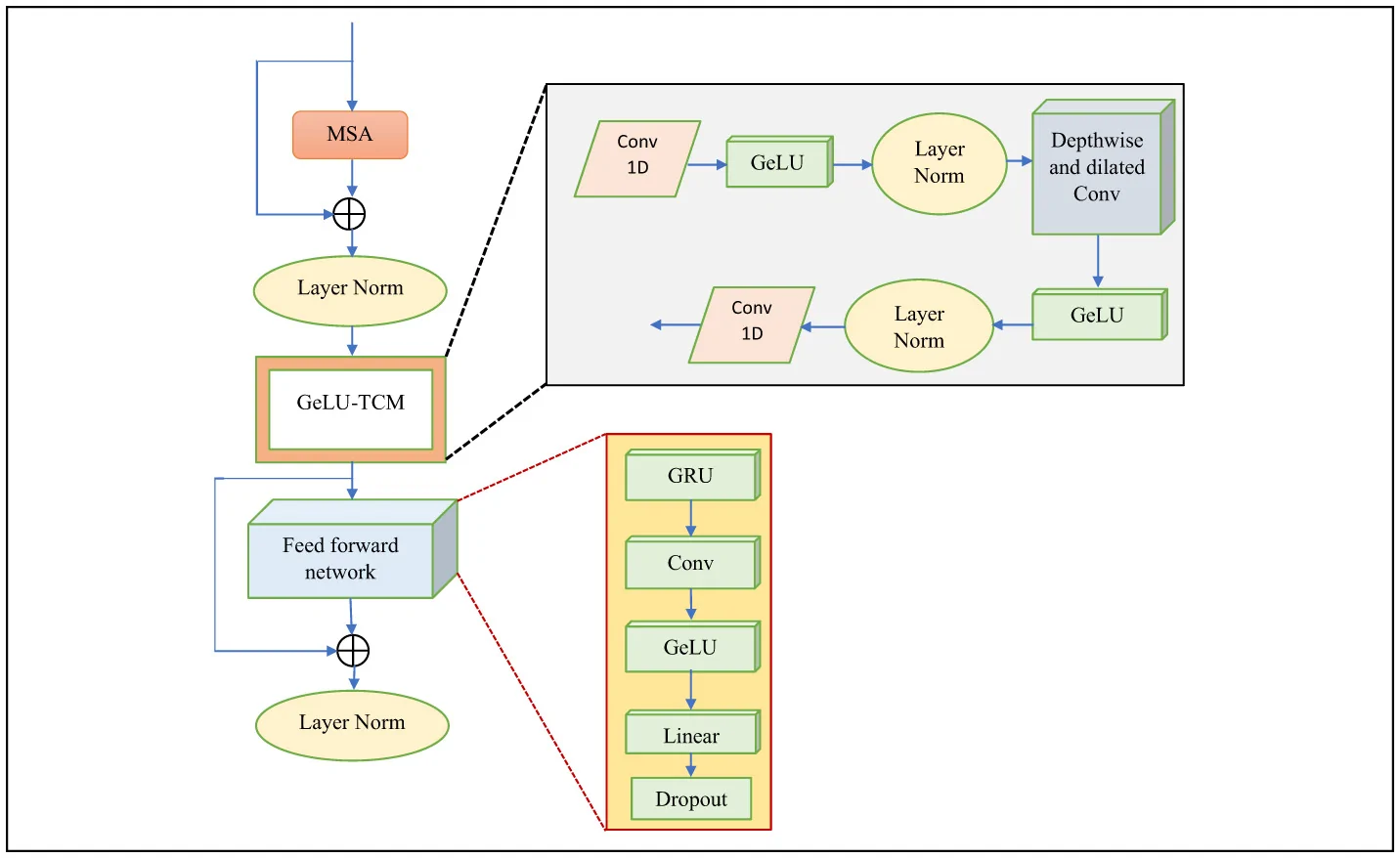
Layered architecture of GeLU-TTCB.

The global context is captured from each patch embedding in the MSA layer. Afterward, layer normalization (LayerNorm) is applied, normalizing the input to each sub-layer. This helps to stabilize and accelerate the training. Following that, a GeLU-TCM is proposed. This GeLU-TCM consists of layers, such as convolutional (conv), GeLU activation, layer normalization, depth-wise and dilated conv layers. The GeLU Activation is provided in Equation 7.

The GeLU activation function is defined by,


$$\begin{array}{l} GeLU\left(j\right)=j*\phi \left(j\right) \end{array}$$ (7)

Here, ϕ(*j*) denotes the Gaussian cumulative distribution function and *j* is the input. For fast computation, the GeLU activation function is provided in Equation 8.


$$\begin{array}{l} GeLU\left(j\right)=0.5j\left(1+\tanh\left[\sqrt{\frac{2}{\pi }}\left(j+0.044715j^{3}\right)\right]\right) \end{array}$$ (8)

The output of the normalization layer passes through the GELU-TCM layers. This module is utilized in the transformer to learn long-term dependencies in the input from the encoder output. Following that, we have utilized a feed-forward network that includes layers such as GRU, convolution, GeLU, linear, and dropout. The output of the GeLU-TCM passes through the feed-forward network, and finally, a layer normalization is applied to stabilize training. The encoder feature representation is *C*_*LR*_. This transformer output (hidden features) might be a one-dimensional (1D) sequence of embeddings that can be converted to a two-dimensional (2D) feature map for convolution and upsampling, which is known as reshaping.

#### 3.2.3 Decoder

An intuitive solution for segmentation purposes is to upsample the encoded feature representation $C_{LR}\in RG^{\frac{H\times Wd}{P^{2}}\times DM}$ to the full resolution for forecasting the dense output. For recovering the spatial order, the encoder feature's size should be reshaped initially from $\frac{H\times Wd}{P^{2}}$to $\frac{H}{P}\times \frac{Wd}{P}$. Moreover, we have utilized a 1 × 1 convolution to reduce the reshaped feature's channel size to the number of classes. Then the feature map gets up-sampled to full resolution *H*×*Wd* for final segmentation outcome prediction. A Cascaded Upsampler (CUP), which includes several upsampling steps for decoding the hidden feature to output the final segmentation mask.

After reshaping the hidden feature's sequence $C_{LR}\in RG^{\frac{H\times Wd}{P^{2}}\times DM}$ to $\frac{H}{P}\times \frac{Wd}{P}\times DM$, the CUP is instantiated via cascading numerous upsampling blocks to attain the full resolution from $\frac{H}{P}\times \frac{Wd}{P}$ to *H*×*Wd*, where each block contains a 3 × 3 convolution layer, a 2 × upsampling operator, and a ReLU layer correspondingly. This CUP, together with the encoder, forms a U-shaped architecture that facilitates feature aggregation at diverse resolution levels through skip connections. These skip connections from CNN encoders were concatenated at each upsampling level. These intermediate skip connections are integrated with Multi-Scale-Depth-Wise Attention Gate (MS-DAG) modules. In each MS-DAG module, a point-wise conv, depth-wise separable convolution block, dilation convolution, BN, and ReLU layers are concatenated. Finally, the sigmoid activation function is applied, and the output is obtained. Moreover, this MS-DAG module extracts and fuses the feature maps at various scales from the pre-processed image. These feature maps can capture global and local information at diverse scales. In addition, this MS-DAG is integrated with skip connections to enhance the efficient extraction of valuable features. The structure of the MS-DAG module is shown in Figure 4.

**Figure 4:**
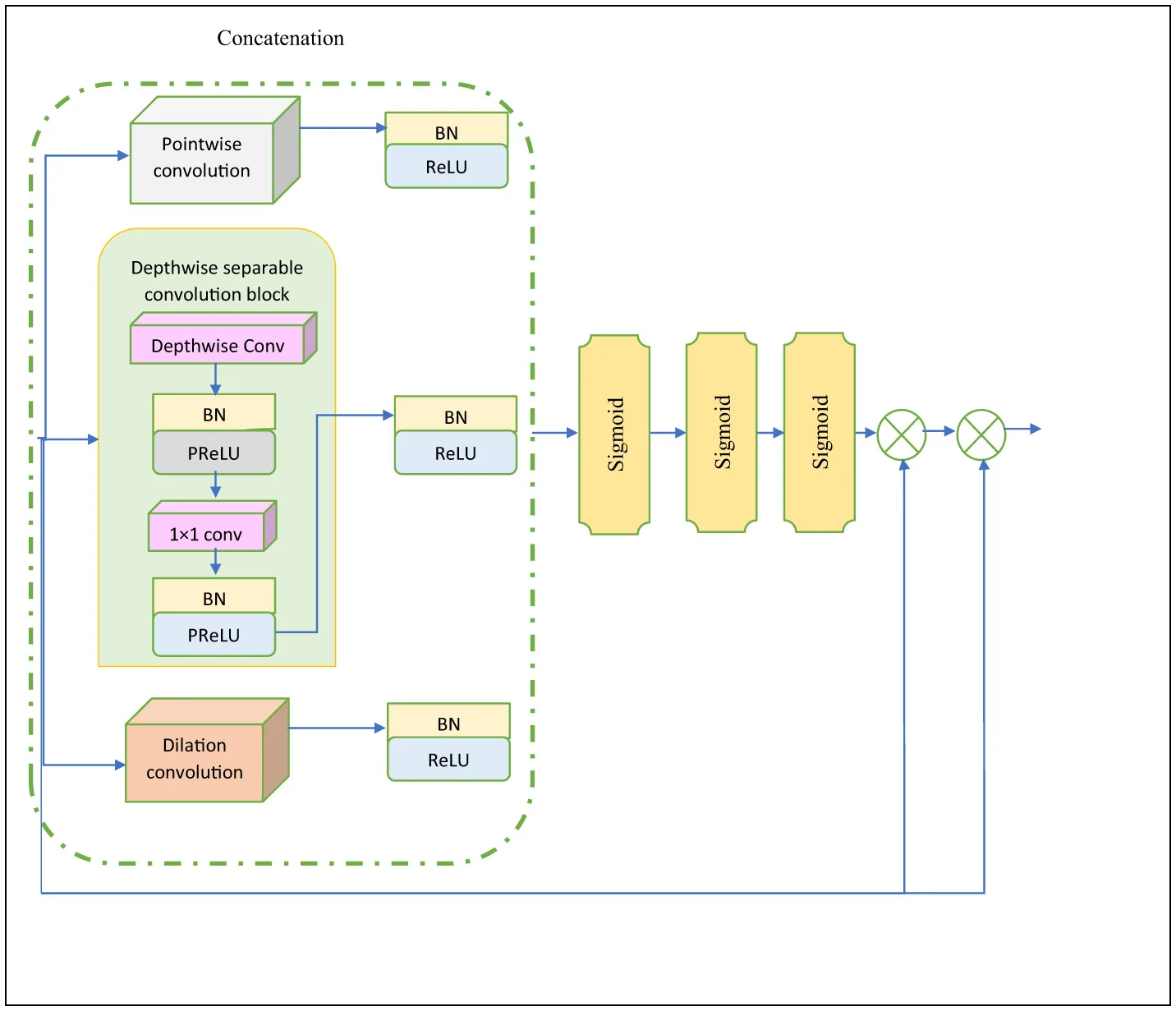
The structure of the MS-DAG module.

Utilizing MS-DAG, residual connections, and GeLU activation in the transformer promotes stable, effective training of the MTransUNet model. Furthermore, this MS-DAG module captures both fine details and global details at different scales, enhancing the segmentation accuracy and robustness of our model. Finally, the segmented image *IM*_*Sg*_ is obtained as output.

### 3.3 Classification and severity estimation

The segmented image *IM*_*Sg*_ is subjected to the classification process. This classification process helps detect whether the image belongs to a normal or abnormal person. If it is abnormal, the severity is detected from the abnormal image, which helps the patient receive treatment without delay. We have utilized a hybrid model named IPCNN-ViT to provide accurate classification and severity estimation, which are described below.

#### 3.3.1 Classification using hybrid IPCNN-ViT

Using the segmented image *IM*_*Sg*_, the classification process is performed with a hybrid model, IPCNN-ViT, to achieve accurate classification. This IPCNN-ViT is the combination of Improved PCNN and Vision Transformer. Here, IPCNN extracts features and generates a feature map, and using this feature map, the classification process is conducted with the ViT model. A PCNN (Ye et al., 2023) is a kind of CNN architecture, where numerous convolutional branches operate in parallel on the segmented image *IM*_*Sg*_. These parallel paths enable the network to extract features at several scales or resolutions, with diverse kinds of filters, and merge them for enhanced performance in classification tasks. However, these parallel convolution branches may extract overlapping or redundant features, which might not effectively improve discriminative power. Different branches may learn redundant features, which can reduce the overall number of identifiable features; this increases the risk of overfitting. To overcome this limitation, we have proposed an IPCNN model, and its architecture is shown in Figure 5.

**Figure 5:**
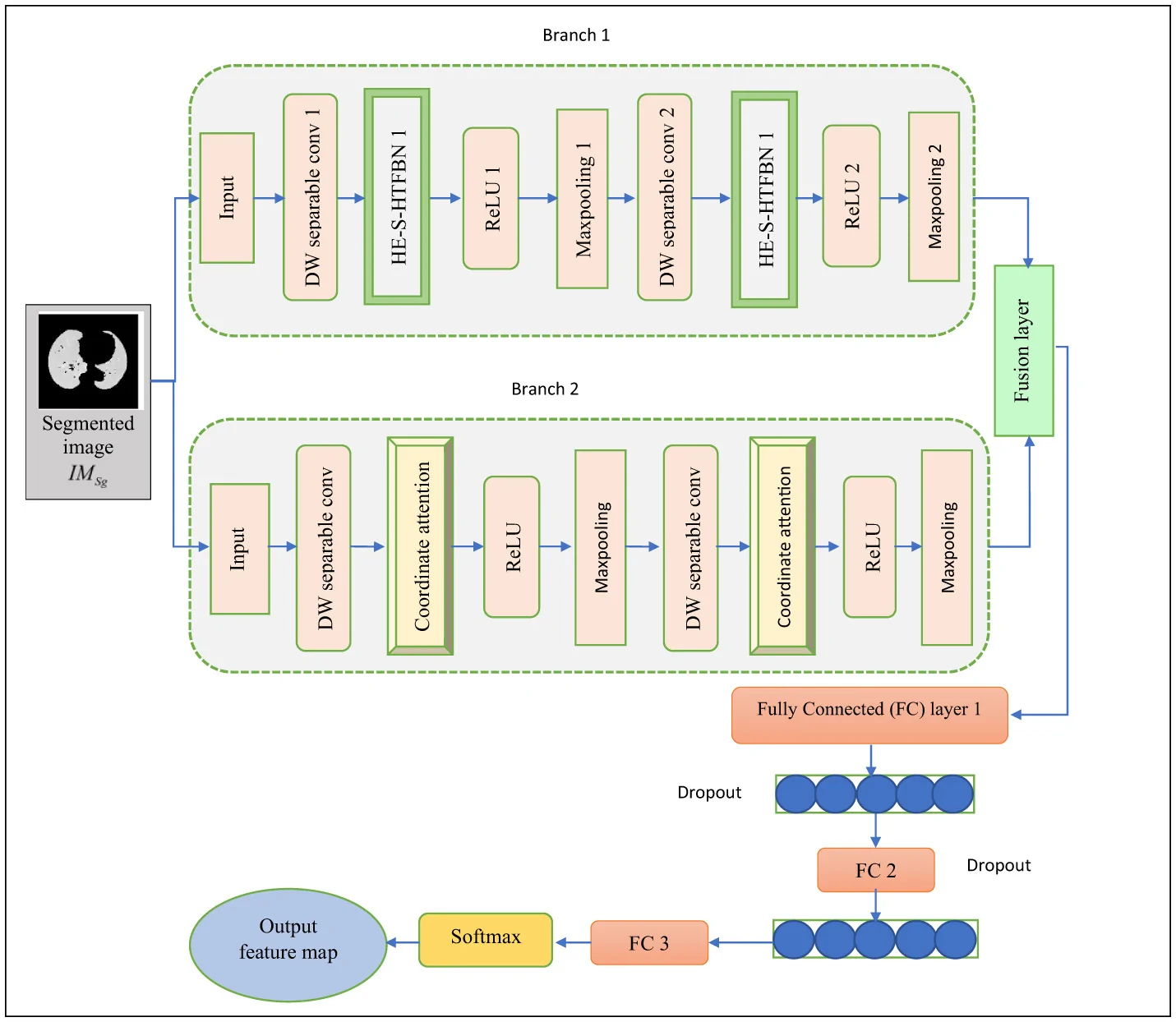
Architecture of IPCNN.

Initially, the *IM*_*Sg*_ is fed into both parallel branches as input. IPCNN's two branches have different layer arrangements. Branch 1 includes layers such as the input layer, depth-wise (DW) separable conv1, HE-S-HTFBN1, ReLU1, Max pooling 1, DW separable conv2, HE-S-HTFBN2, ReLU2, and Max pooling 2. The *IM*_*Sg*_ is given to the input layers, and features are extracted in the DW-separable conv1 layers. These obtained feature vectors go through the proposed HE-S-HTFBN1 layer. This HE-S-HTFBN is a combination of the Swish-HardELiSH activation function and hyperbolic tangent function-based normalization.

A computationally cheaper approximation of the ELiSH activation function is known as the HardELiSH activation function, and that is presented in Equation 9.


$$\begin{array}{l} HE\left(\chi \right)=\left\{\begin{array}{cc} \chi .HardSigmoid\left(\chi \right), & if\chi \geq 0\\ \left(\exp\left(\chi \right)-1\right).HardSigmoid\left(\chi \right), & if\chi <0 \end{array}\right\} \end{array}$$ (9)

where $HardSigmoid\left(\chi \right)=clip\left(\frac{\chi +1}{2},0,1\right)$ and χ is the feature map.

The Swish activation function (Pydimarry et al., 2024) has been considered as a smooth and non-monotonic activation function, which is given in Equation 10:


$$\begin{array}{l} SW\left(\chi \right)=\chi .sigmoid\left(\chi \right)=\left[\chi *\frac{1}{1+e^{-\chi }}\right] \end{array}$$ (10)

The Swish-Hard ELiSH activation function is given in Equation 11:


$$\begin{array}{l} S\left(\chi \right)=\left\{\begin{array}{cc} \chi *\max\left(0,\min\left(1,\frac{\chi +1}{2}\right)\right); & \chi \geq 0\\ SW\left(\chi \right)*\max\left(0,\min\left(1,\frac{\chi +1}{2}\right)\right); & \chi <0 \end{array}\right\} \end{array}$$ (11)

The hyperbolic tangent function-based normalization is provided in Equation 12:


$$\begin{array}{l} f\left(\chi \right)=\frac{1-e^{\left(\frac{\mu -\chi }{\sigma }\right)}}{1+e^{\left(\frac{\mu -\chi }{\sigma }\right)}} \end{array}$$ (12)

where μ and σ are the mean and standard deviation of the feature map χ and hyperbolic tangent function-based normalization is *f*(χ).

In the HE-S-HTFBN layer, the normalization process is performed, and is mathematically expressed as in Equation 13.


$$\begin{array}{l} HE-S-HTFBN_{\left(\chi \right)}=\left[\frac{f\left(\chi \right)-\mu^{k}\left(f\left(\chi \right)\right)}{\sigma^{k}f\left(\chi \right)+\varepsilon }*S\left(\chi \right)\right] \end{array}$$ (13)

where μ^*k*^(*f*(χ)) is the mean of HTFBN output which can be calculated utilizing Equation 14, and σ^*k*^(*f*(χ)) is the variance of HTFBN output, and that can be calculated utilizing Equation 15, *S*(χ) = *Swish*−*HardELiSH*(χ) and ε is a small constant to avoid division by zero. Also, *i* denotes the feature dimension, that is, *i* = 1, 2, ..*mn*.


$$\begin{array}{l} \mu^{k}\left(f\left(\chi \right)\right)=\frac{1}{mn}\overset{mn}{\underset{i=1}{\sum }}f\left(\chi \right) \end{array}$$ (14)

$$\begin{array}{l} \sigma^{k}\left(f\left(\chi \right)\right)=\frac{1}{mn}\overset{mn}{\underset{i=1}{\sum }}\left[f\left(x\right)-\mu \left(f\left(\chi \right)\right)\right] \end{array}$$ (15)

Afterward, the normalized output passes through the ReLU and Maxpooling layers, where the spatial dimensions are reduced, and the resulting downsampled feature map is used as input to the DW separable conv2 layer. After passing through the HE-S-HTFBN2, ReLU2, and Max pooling 2 layers, a downsampled feature map is finally obtained.

This proposed HE-S-HTFBN helps stabilize training by better adapting normalization and improving training. Coordinate attention (CA) embeds coordinate information into channel attention, enhancing the model's ability to recognize spatially relevant features for lung cancer classification. This HE-S-HTFBN and DW conv help prevent overfitting, increase model performance, and improve robustness.

Similarly, branch 2 includes layers like input, DW separable conv1, Coordinate Attention (CA), ReLU1, maxpooling1, DW separable conv2, Coordinate Attention (CA), ReLU, and maxpooling2.

To the input layer, *IM*_*Sg*_ is provided, and from that image, features are extracted in the DW separable conv1 layer and are given to the CA module as input. In computer vision, AMs are widely used. We have utilized a CA mechanism that embeds location information into channel attention to capture feature-location dependencies by integrating AMs with diverse horizontal and vertical orientations. The structure of the CA module is shown in Figure 6.

**Figure 6:**
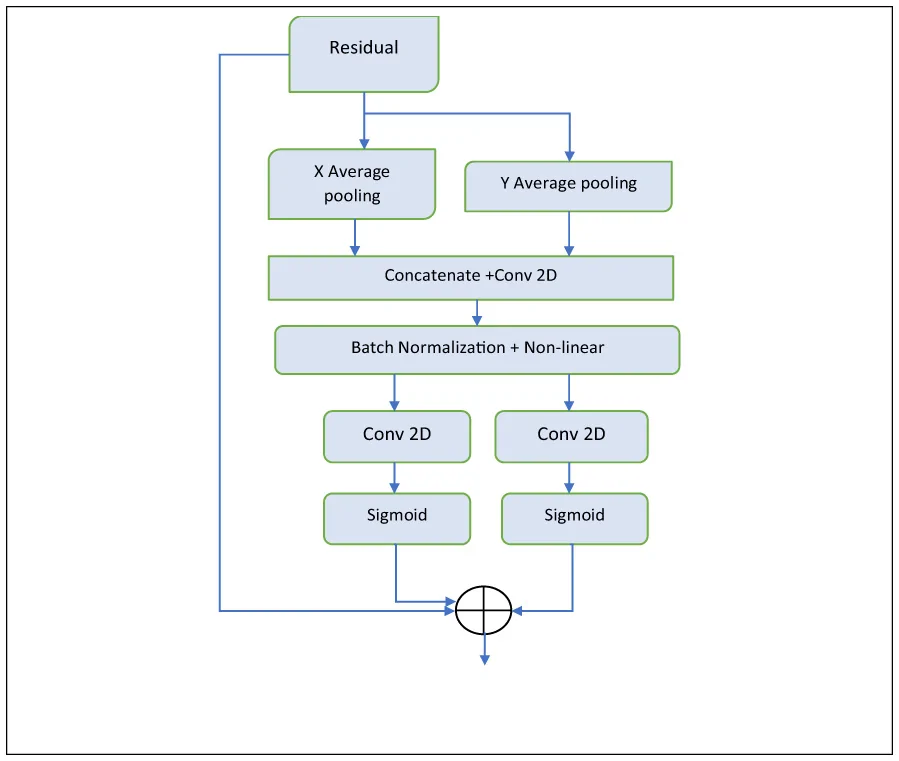
Structure of CA module.

The global pooling has been decomposed into two feature encoding operations. The coordinate information is embedded. Initially, for input *IM*_*Sg*_, each channel gets encoded along the horizontal as well as vertical coordinate directions with the utilization of pooling kernels having dimensions (*Ht*, 1) and (1, *Wdt*), so that the 1D features attained in the horizontal direction are provided in Equation 16:


$$\begin{array}{l} Z_{q}^{ht}\left(ht\right)=\frac{1}{Wdt}\left(\underset{0\leq Q\leq wd}{\sum }x_{q}\left(ht,J\right)\right) \end{array}$$ (16)

Similarly, the 1D features attained in the horizontal direction are provided in Equation 17


$$\begin{array}{l} Z_{q}^{wd}\left(ht\right)=\frac{1}{Ht}\left(\underset{0\leq J\leq Ht}{\sum }x_{q}\left(J,wd\right)\right) \end{array}$$ (17)

The coordinate embedding process, Equations 16, 17 can be utilized effectively to attain the global receptive field along with encoding precise location information. To effectively utilize the resulting representations, a second transformation, CA, is designed. The two feature maps, which are generated by the previous module, are cascaded initially and afterward transformed *Fn*_1_ utilizing a shared 1 × 1 convolution, as provided in Equation 18.


$$\begin{array}{l} fn=\delta \left(Fn_{1}\left(\left[Z^{ht}\Theta Z^{wt}\right]\right)\right) \end{array}$$ (18)

where [Θ] denotes the concatenation operation and is denoted by a non-linear activation function. The horizontal as well as vertical results were stitched by *Fn*_1_ and the intermediate feature mapping is *fn*. A hyperparameter that controls the module's size *r*. Subsequently, *fn*is divided into two separate tensors *fn*^*ht*^∈*R*^*Ct*/*r*×*Ht*^and *fn*^*wd*^∈*R*^*Ct*/*r*×*Wdt*^along the spatial dimension. Afterward, the feature maps *fn*^*ht*^and *fn*^*wd*^were converted to the same number of channels as the input, utilizing 1 × 1 convolutions *Fn*_*ht*_ and *Fn*_*wd*_, resulting in Equations 19, 20.


$$\begin{array}{l} gt^{ht}=\sigma \left(Fn_{ht}\left(fn^{ht}\right)\right) \end{array}$$ (19)

$$\begin{array}{l} gt^{wd}=\sigma \left(Fn_{wd}\left(fn^{wd}\right)\right) \end{array}$$ (20)

where σ symbolizes the sigmoid function. Afterward, the expansion of *gt*^*ht*^and *gt*^*wd*^has been conducted as attention weights. Finally, the input–output residual concatenation operation is performed. The CA module's output can be expressed as in Equation 21.


$$\begin{array}{l} CA\left(Q,J\right)=x_{q}\left(Q,J\right)\times gt_{q}^{ht}\left(Q\right)\times gt_{q}^{wd}\left(J\right) \end{array}$$ (21)

This CA module's output passes through the ReLU1 and Maxpooling1 layers, where the spatial dimensions are reduced, and the downsampled feature map is given to the DW separable conv2 layers. The output of this DW separable conv2 layer passes through the CA module, ReLU, and Maxpooling layers, yielding a downsampled feature map.

The generated downsampled feature maps from both branches of IPCNN are fused in the fusion layer, and then given to the fully connected layers as input, where the feature maps are generated. There are three fully connected layers, and dropout is applied before each one. Finally, a Softmax activation is applied to these feature maps, and the downsampled feature map is obtained as the output.

Afterward, the feature map obtained from IPCNN is used as input to the ViT for classification. An architecture that applies the transformer architecture to computer vision tasks is named ViT (Okolo et al., 2022). Here, we have utilized ViT for classification because it can achieve better performance, especially in image classification tasks. Moreover, this ViT has efficient transfer learning capabilities and can handle large-scale datasets effectively.

This ViT divides the feature maps into patches, which are then treated as a sequence of tokens. The ViT-based classification process includes six steps, which are summarized below.

(1) Dividing the feature maps into patches: first, feature maps are divided into fixed-size patches. Afterward, each patch gets flattened into a 1D vector.
(2) Linear embedding of patches: each flattened patch is linearly embedded into a vector of fixed-size.
(3) Adding positional embeddings: for each patch embedding, positional embeddings are added to retain spatial information. This helps to comprehend each patch's relative position within the *IM*_*Sg*_.
(4) Class token: to the embedded patch's sequence, a learnable classification token has been prepended. From all patches, the information is aggregated using this token, which can be ultimately utilized for classification.
(5) Transformer encoder: the vector's sequence (class token + embedded patches) goes through a series of transformer encoder layers. Each layer includes multi-head self-attention and Multi-layer Perceptron (MLP) blocks. Each encoder layer processes the input sequence, and an output sequence of the same length and dimension is produced. This self-attention mechanism enables each patch to attend to all other patches, facilitating the model's capture of long-range dependencies and interactions between patches.
(6) Classification head: the class token's final hidden state is utilized for classification. To the class token's output, a fully connected layer has been applied, which predicts the class probabilities as normal (0) or abnormal (1).

#### 3.3.2 . Severity estimation using hybrid IPCNN-ViT

After classifying the normal and abnormal images, the abnormal images (people with lung cancer) are fed into the same hybrid IPCNN-ViT for severity estimation. Therefore, this IPCNN-ViT classifier classifies the severity level of lung cancer into four classes: 0 = Mild, 1 = Moderate, 2 = Severe, and 3 = More Severe. Based on these severity levels, patients receive treatment. Severity classification was performed in two hierarchical stages based on the consensus malignancy score (mean of up to four radiologists' ratings on the LIDC-IDRI 1–5 scale). In Stage 1, scans were categorized as Normal (no significant nodule, score = 1) or Abnormal (nodule present, score ≥ 2). In Stage 2, Abnormal cases were further stratified into four severity classes: mild, moderate, severe, and very severe by dividing the residual score range (2–5) into four equal-width intervals. Moreover, a clear explanation is mentioned in Section 3.3.2.

## 4 Result and discussion

### 4.1 Simulation procedure

The Proposed Lung Cancer Classification and Severity Estimation Using Vision Transformer-IPCNN with Enhanced U-Net Segmentation was implemented and simulated using Python. The system configuration included an AMD Ryzen 5 3450U processor with Radeon Vega Mobile Graphics at 2.10 GHz and 16 GB of RAM (13.9 GB usable) on a 64-bit, x64-based operating system. The proposed framework was developed using TensorFlow and Python-based libraries, including PyDICOM, SciPy, Scikit-learn, OpenCV, and NumPy. The analysis of Lung Cancer Classification and Severity Estimation was performed using the LIDC-IDRI Dataset (Armato et al., 2011) and the LUNA16 Dataset (used for cross-validation only).

### 4.2 Dataset 1 description: LIDC-IDRI dataset

The LIDC-IDRI dataset is a publicly available collection of thoracic CT scans with annotated lung lesions used to develop and evaluate computer-assisted diagnosis (CAD) methods for lung cancer. It includes 1,018 cases, each with a CT scan and an XML file containing annotations from four expert radiologists. A two-phase annotation process, blinded and unblinded, was used to accurately identify lung nodules of different sizes, ensuring comprehensive labeling, without requiring forced consensus.

The proposed implementation framework uses 2,241 data points with binary classes of 0: non-Tumor (1,117 data points) and 1: tumor (1,124 data points). The severity classes have 1,124, with multi-classes of 0–3 (0 = Mild, 1 = Moderate, 2 = Severe, 3 = More Severe), with data: Class 0: 394, Class 1: 256, Class 2: 236, and Class 3: 238. The training and testing details of the dataset split-up are tabulated in Table 2.

**Table 2:** Training and testing split-up details of dataset.

Training percentage (%)	Training samples	Non-tumor in train	Tumor in train	Testing samples	Non-tumor in test	Tumor in test
60	1,344	670	674	897	447	450
70	1,568	782	786	673	335	338
80	1,792	893	899	449	224	225
90	2,016	1,005	1,011	225	112	113

### 4.3 Dataset 2 description: LUNA16 dataset (used for cross-validation only)

The LUng Nodule Analysis 2016 (LUNA16) dataset (Setio et al., 2017) is an open-source collection of thoracic computed tomography (CT) images designed for lung nodule analysis and classification. This dataset includes 888 CT scans, of which 589 are cancer cases and 299 are non-cancer cases. In this study, a total of 445 patient samples were selected, of which 306 patients had cancer and 139 did not have cancer, to assess the proposed method for lung cancer classification and severity evaluation. The chosen CT images were fed into the pre-processing stage, lung region segmentation, and then the classification to distinguish cancer and non-cancer cases.

### 4.4 Evaluation of lung cancer classification and severity prediction

Under the concept of lung cancer classification and severity estimation using Vision Transformer-IPCNN, we are evaluating which classifier model safely and accurately identifies lung cancer cases. With the support of these metrics, it prohibits the different aspects of how the system detects cancer patients and non-cancer cases. Here, we have taken CNN + Mask R-CNN (Akintola et al., 2025) and PSSO-based DL (Choudhury et al., 2023) as base models to showcase our implemented enhancement.

Figures 7, 8 clearly present the comparison graph of the classifier model's detection efficiency using metrics. Figure 7b shows the accuracy ranges of the classifiers, implying the overall correctness of the models in predicting positive and negative cases. Figure 7a categorizes the sensitivity scores of the classifiers, indicating how they detect actual cancer cases. Among these two cases, the baseline IPCNN classification approach has yielded lower accuracy of 93.18% in 70% and 95.86% in 90% of the data in the training stages, and a sensitivity of 94.93% in 80% and 96.09% in 90%. The LCCSE-IPCNN-ViT approach has enhanced its classification framework by designing the IPCNN-ViT to achieve higher accuracy of 96.5% in 70% and 98.8% in 90%, followed by a sensitivity of 98.66% in 90% of training variation. The specificity and precision case measures calculate how many cases predicted as lung cancer are correct and how many non-cancer cases are identified, as graphed in Figures 7c, d. In these cases, the IPCNN-ViT classifier has achieved the highest precision and specificity of 98.95% across 90% of the training data variation.

**Figure 7:**
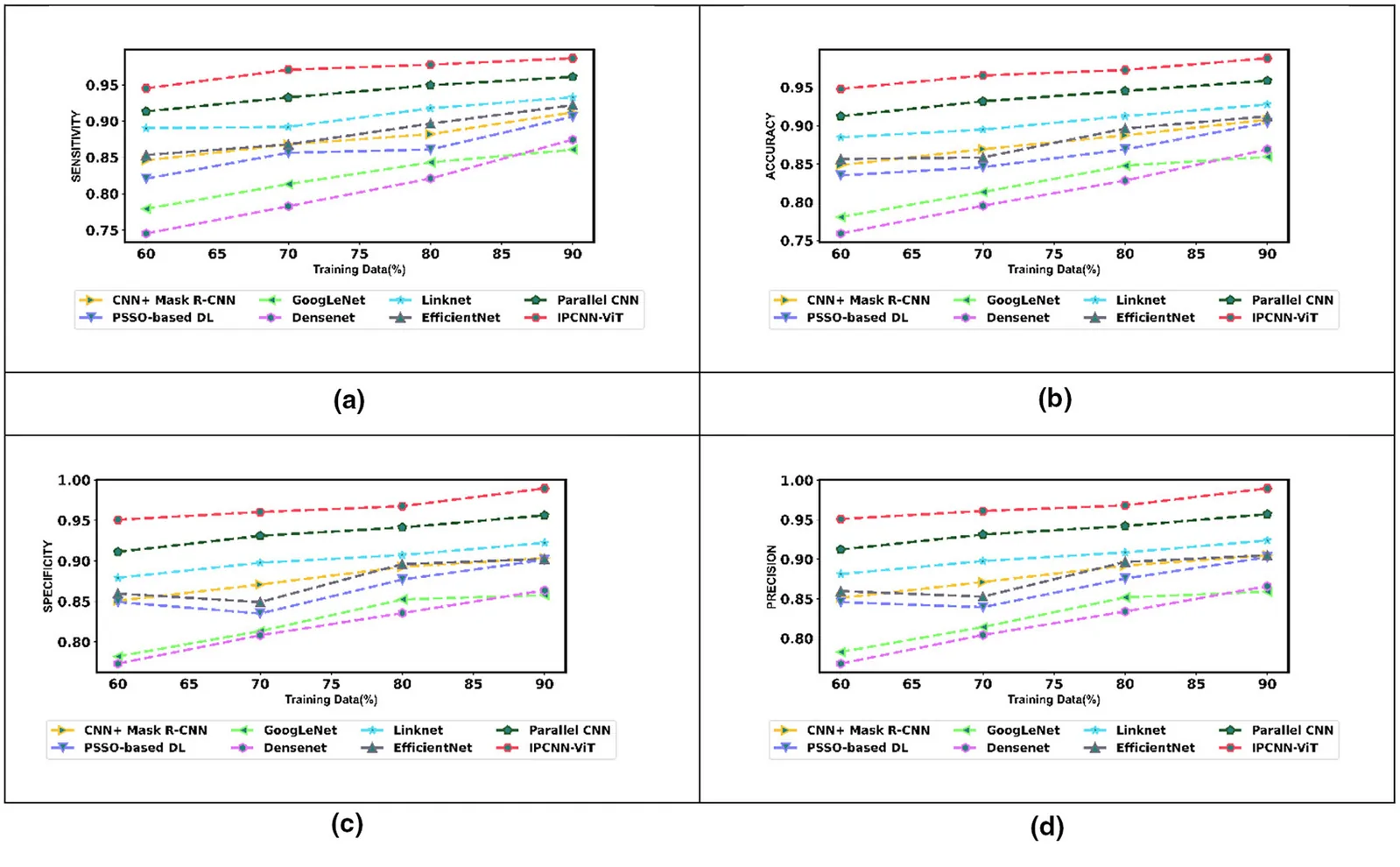
Reliability analysis of lung cancer classification model (in positive cases). **(a)** Sensitivity; **(b)** Accuracy; **(c)** Specificity; **(d)** Precision.

**Figure 8:**
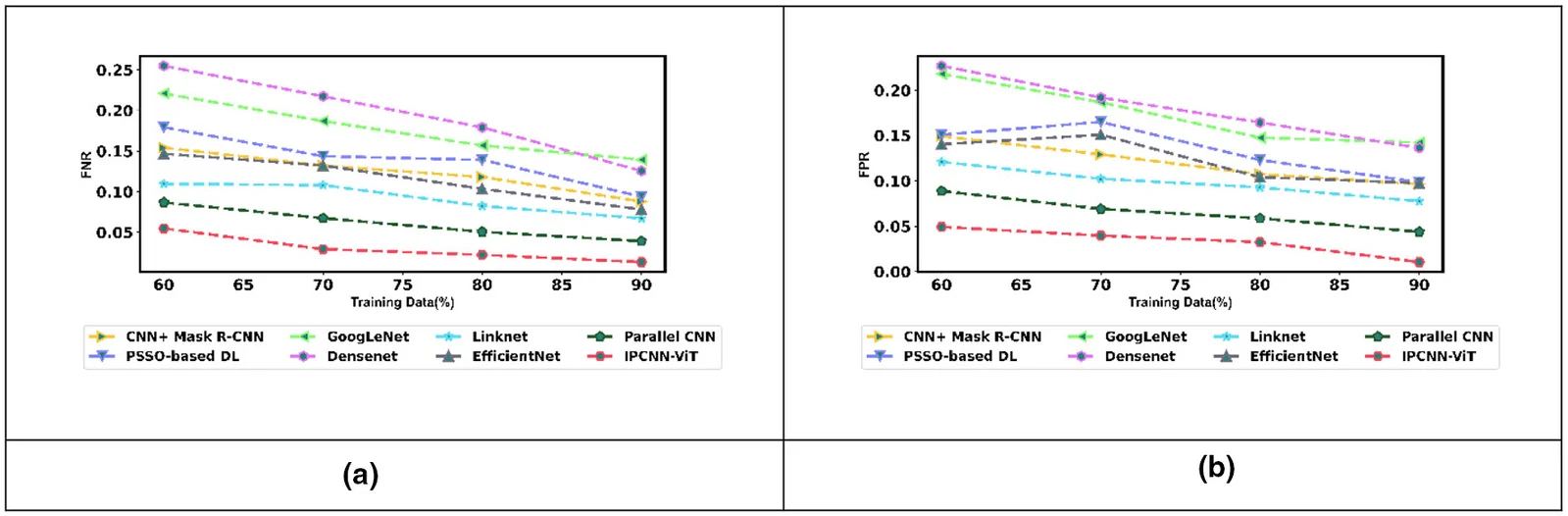
Reliability analysis of lung cancer classification model (in negative cases). **(a)** FNR; **(b)** FPR.

The following section presents the scores of baselines and IPCNN-ViT classifier models under negative estimation cases, using the support of false-positive rate (FPR) and false-negative rate (FNR) metrics. These cases should be reduced to verify that the classifier accurately detects lung cancer patients without misclassification. Based on the observations, the baseline DenseNet classifier achieved the highest FNR and FPR across the data training variation levels. Especially in 60 of FNR estimation, the DenseNet has marked the range of 25.4–12.54% at 90% (Figure 8a), and likely in FPR, it achieved 16.44% at 80% and 13.63% at 90% (as shown in Figure 8b). The IPCNN-ViT approach has reduced the baseline mislabeling activity under the classification of lung cancer to express the extremely minimal FNE and FPR of 1.33 and 1.04%, respectively, under 90% (training set).

The validation is performed in other cases to assess how the lung cancer detection model is working. F-measure checks whether the model balances the detection of lung cancer cases while keeping false classifications low. Consequently, the Matthews Correlation Coefficient (MCC) and Negative Prediction Value (NPV) are emphasized to ensure model prediction quality. This metric outcome is displayed in Figure 9 with the sub-divisions. The IPCNN-ViT approach has resulted in F-measure and NPV scores of nearly 98 and 97%, respectively, across 90% of training variations.

**Figure 9:**
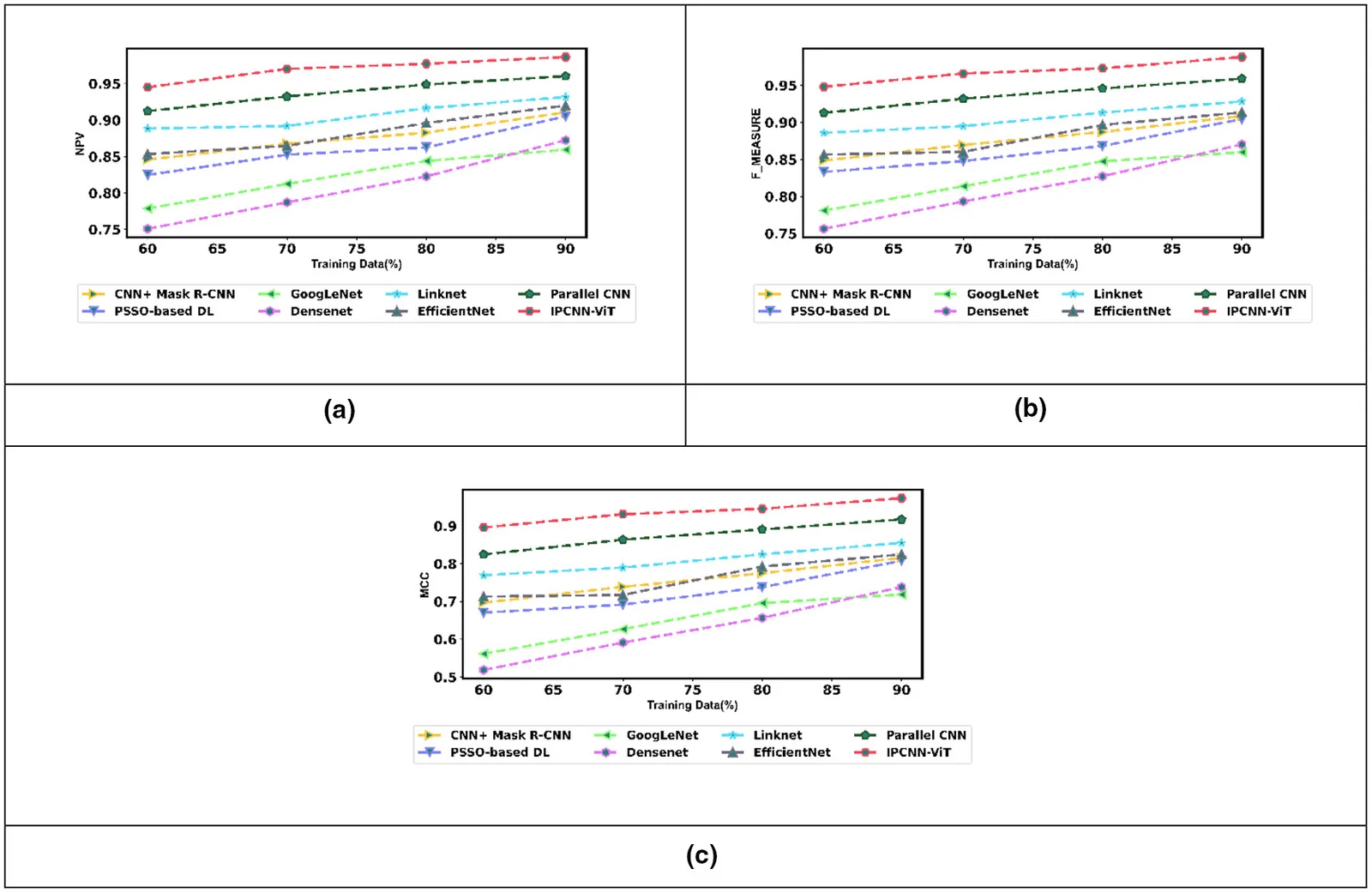
Reliability analysis of lung cancer classification model (in other cases). **(a)** NPV; **(b)** *F*-measure; **(c)** MCC.

#### 4.4.1 Severity classification

The severity classification outcome comparison between the baseline and IPCNN-ViT classifier is summarized and visualized in Figure 10. This type of classification is helpful for quickly examining which areas the model is strong in and where it is weak in lung cancer detection. For that, we will evaluate using various measures, categorized as mild, moderate, severe, and more severe. Here, we categorize the positive and negative estimates for both approaches in the sub-categorization of Figures 10a, b. The overall observation summarizes that the LCCSE-IPCNN-ViT model maintains its classification correctly, with maximal accuracy and precision of 98% and 96.1%, respectively. At a 90% training data proportion, misclassification of lung cancer results in lower metric scores of FNR and FPR by 3.8 and 1.29%, respectively.

**Figure 10:**
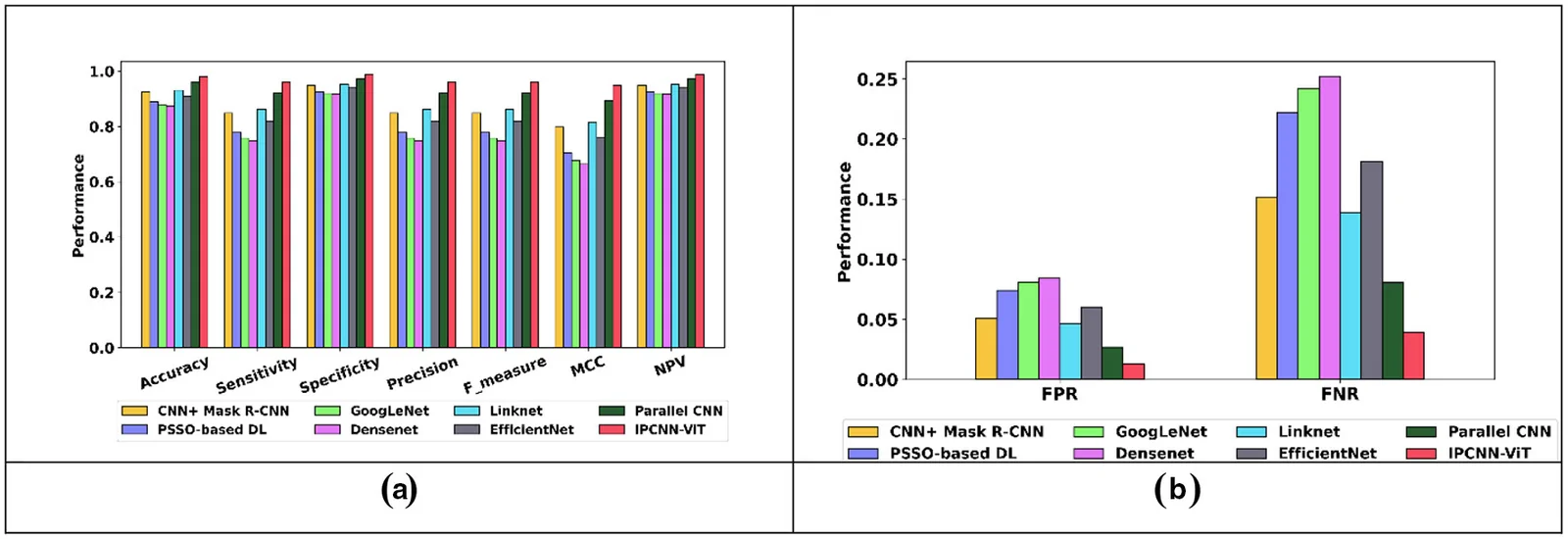
Severity classification outcome of LCCSE-IPCNN-ViT approach. **(a)** Severity classification of positive measures; **(b)** Severity classification of negative measures.

### 4.5 Ablation experimental outcome

Within this concept, we undertook ablation classification to assess their essentiality across lung cancer classifications and severity ranges. Table 3 summarizes the importance by processing methods, namely without pre-processing and segmentation, and conventional frameworks such as Wiener (pre-process), transUNET, and vision transformation with the LCCSE-IPCNN-ViT framework. Without the initial stages of pre-processing and segmentation, the models are unable to accurately detect lung cancer and predict their severity levels because these stages play a key role in normalizing the data, highlighting affected regions, and helping the Vision Transformer-IPCNN learn important features more effectively. Regarding this, they achieved lower accuracies of 90.3 and 91.2% across 80% of the training sets. Moreover, the conventional Wiener and transUNET frameworks have faced limitations in the pre-processing step due to the high frequency of blurred edges and fine details. Along with this, the segmented approach has challenges due to the loss of fine-grained local details. These difficulties are leading to baseline models with lower accuracy: 93.2% (pre-processed) and 92.4% for conventional transUNET. The LCCSE-IPCNN-ViT approach has introduced an enhanced version of the pre-processing and segmentation framework with an improved PCNN and Vision Transformer, boosting detection reliability to 97.3%.

**Table 3:** Ablation outcome of LCCSE-IPCNN-ViT approach (over 80% of training stage).

Metrics	Without pre-processing	Conventional Wiener pre-process	Conventional transUNET	Without segmentation	Conventional ViT	LCCSE-IPCNN-ViT
Specificity	0.908	0.931	0.924	0.912	0.940	0.968
Accuracy	0.903	0.932	0.924	0.912	0.941	0.973
Sensitivity	0.899	0.933	0.924	0.912	0.943	0.978
F-measure	0.902	0.932	0.923	0.911	0.941	0.973
MCC	0.872	0.899	0.889	0.878	0.904	0.945
NPV	0.901	0.934	0.925	0.913	0.943	0.977
Precision	0.906	0.930	0.922	0.911	0.939	0.968
FPR	0.092	0.069	0.076	0.088	0.060	0.032
FNR	0.101	0.067	0.076	0.088	0.057	0.022

### 4.6 Statistical study

The statistical assessment review is based on accuracy, which is tabulated in Table 4. Among these observations, the baseline classification approaches achieve the lowest scores due to their insufficiency in detecting lung cancer. In particular, the DenseNet model has achieved a lower mean accuracy of 81.3%, a median of 81.2%, a minimum of 75.9%, and a maximum of 86.9%. Similarly, the GoogLeNet model has achieved mean and maximum accuracies of 82.5 and 85.9%. Further distinguishing the baseline Parallel CNN from IPCNN-ViT, the IPCNN-ViT has delivered boosted efficiency ranges with a mean accuracy of 96.9% and maximal and minimal ranges of 98.8 and 94.8%, respectively, to better adapt to normalization.

**Table 4:** Statistical results of the IPCNN-ViT classifier (accuracy).

Metrics	CNN + Mask R-CNN	PSSO-based DL	GoogLeNet	DenseNet	Linknet	EfficientNet	Parallel CNN	IPCNN-ViT
Mean	0.878	0.863	0.825	0.813	0.905	0.881	0.937	0.969
Median	0.878	0.857	0.830	0.812	0.904	0.877	0.939	0.969
Standard	0.022	0.026	0.031	0.041	0.016	0.024	0.017	0.014
Minimum	0.848	0.835	0.781	0.759	0.885	0.857	0.912	0.948
Maximum	0.908	0.904	0.859	0.869	0.928	0.912	0.959	0.988

### 4.7 Significance analysis of model results

In this section, we carry out different statistical testing approaches, namely Friedman, Wilcoxon, and Kruskal, to validate the model's reliability. These tests are used to determine whether there are any differences in results, such as between multiple models, modifications, and across different severity groups. Each testing outcome is obtained by comparing the IPCNN-ViT with the baseline and is expressed as a rank-based *p*-value. Table 5 summarizes the outcome of the Friedman test, which is performed among three or more models on the same dataset, and Table 6 reflects the Wilcoxon outcome, which is obtained by comparing two related sets of results. For both tests, the alpha value is set to 0.05. Under this interpretation, if the *p*-value < alpha value, it mentioned that there are significant differences, and if the *p*-value > alpha value, it is highlighted that there are no significant differences. In the Friedman test, the DenseNet model with IPCNN-ViT has a *p*-value of 0.0289, and IPCNN-ViT with Linknet has a *p*-value of 0.0152. These cases show that the *p*-value is less than the alpha value, so there are significant differences among these models, and the baseline PCNN model has a Friedman test *p*-value of 0.0714 and a Wilcoxon test *p*-value of 0.0755, indicating there is no significant difference.

**Table 5:** Friedman test of IPCNN-ViT vs. baseline approaches.

Proposed vs.	Friedman chi-square *p*-value (alpha = 0.05)
CNN + Mask R-CNN	0.0305
PSSO-based DL	0.0163
GoogLeNet	0.0850
DenseNet	0.0289
Linknet	0.0152
EfficientNet	0.0032
Parallel CNN	0.0714

**Table 6:** Wilcoxon test of IPCNN-ViT vs. baseline approaches.

Proposed vs.	Wilcoxon test (alpha = 0.05)
CNN + Mask R-CNN	0.0205
PSSO-based DL	0.0914
GoogLeNet	0.0294
DenseNet	0.0002
Linknet	0.0006
EfficientNet	0.0536
Parallel CNN	0.0755

Table 7 outlines the Kruskal test results for the proposed framework, which compares three or more independent groups to analyze how the models perform across different severity levels of lung cancer. If the *p*-value ≤ the alpha value, it states that at least one group's performance is significantly different; if the *p*-value > the alpha value, it indicates that there is no significant difference and all groups perform similarly. Here, the alpha values are assumed to be 0.1. In that, the baseline DenseNet and Parallel CNN have acquired higher *p*-values of 0.1675 and 0.1988, indicating there is no significant difference. Among all these three statistical tests, the LCCSE-IPCNN-ViT model vs. baseline models have prohibited significant differences in the classification of lung cancer and severity to prove it.

**Table 7:** Kruskal test of IPCNN-ViT vs. baseline approaches.

Proposed vs.	Kruskal *p*-value (alpha = 0.1)
CNN + Mask R-CNN	0.0765
PSSO-based DL	0.0007
GoogLeNet	0.0786
DenseNet	0.1675
Linknet	0.0015
EfficientNet	0.0094
Parallel CNN	0.1988

### 4.8 Assessment of image quality metrics

Throughout this portion of the assessment, the Peak Signal-to-Noise Ratio (PSNR) and Structural Similarity Index Measure (SSIM) metrics are used to evaluate the pre-processed image quality. PSNR shows how there constructed (pre-processed) image retains the original details, and the SSIM measures how similar the two images are in terms of structure and contrast as defined in Equations 22, 23. In both cases, the model that achieves a higher accuracy score confirms that it has preserved the important and meaningful features to detect lung cancer more effectively.


$$\begin{array}{c} PSNR=10.\log_{10}\left(\frac{MA{X_{B}}^{2}}{MSE}\right) \end{array}$$ (22)

$$\begin{array}{c} SSIM\left(B,Q\right)=\frac{\left(2_{\mu B\mu Q}+T_{1}\right)\left(2_{\sigma BQ}+T_{2}\right)}{\left({\mu_{B}}^{2}+{\mu_{Q}}^{2}+T_{1}\right)\left({\sigma_{B}}^{2}+{\sigma_{Q}}^{2}+T_{2}\right)} \end{array}$$ (23)

Table 8 details the quality metric ranges among the baseline filtering approaches and IWF approaches. The mean filtering technique achieved the lowest PSNR and SSIM scores of 31.656 and 0.903, indicating that the pre-processed images have distortion. The conventional Wiener filter achieved a higher PSNR of 40.329 and an SSIM of 0.943 than the Mean Filter. However, it is facing issues in its pre-processing stage due to high-frequency components, which lead to a reduction in sharpness. To overcome this issue, the LCCSE-IPCNN-ViT model employs the Wiener Filtering Method (IWF) to maintain sharp boundaries with fine details. As part of this, it achieves the maximum PSNR and SSIM of 43.499 and 0.963, respectively.

**Table 8:** PSNR and SSIM metric validation of filtering frameworks.

Filters	PSNR	SSIM
Gaussian filter	34.218	0.930
Mean filter	31.656	0.903
Conv Wiener	40.329	0.943
IWF	43.499	0.963

#### 4.8.1 Pre-processing outcome

The pre-processed images of various baseline filtering techniques and IWF are visualized in Figure 11.

**Figure 11:**
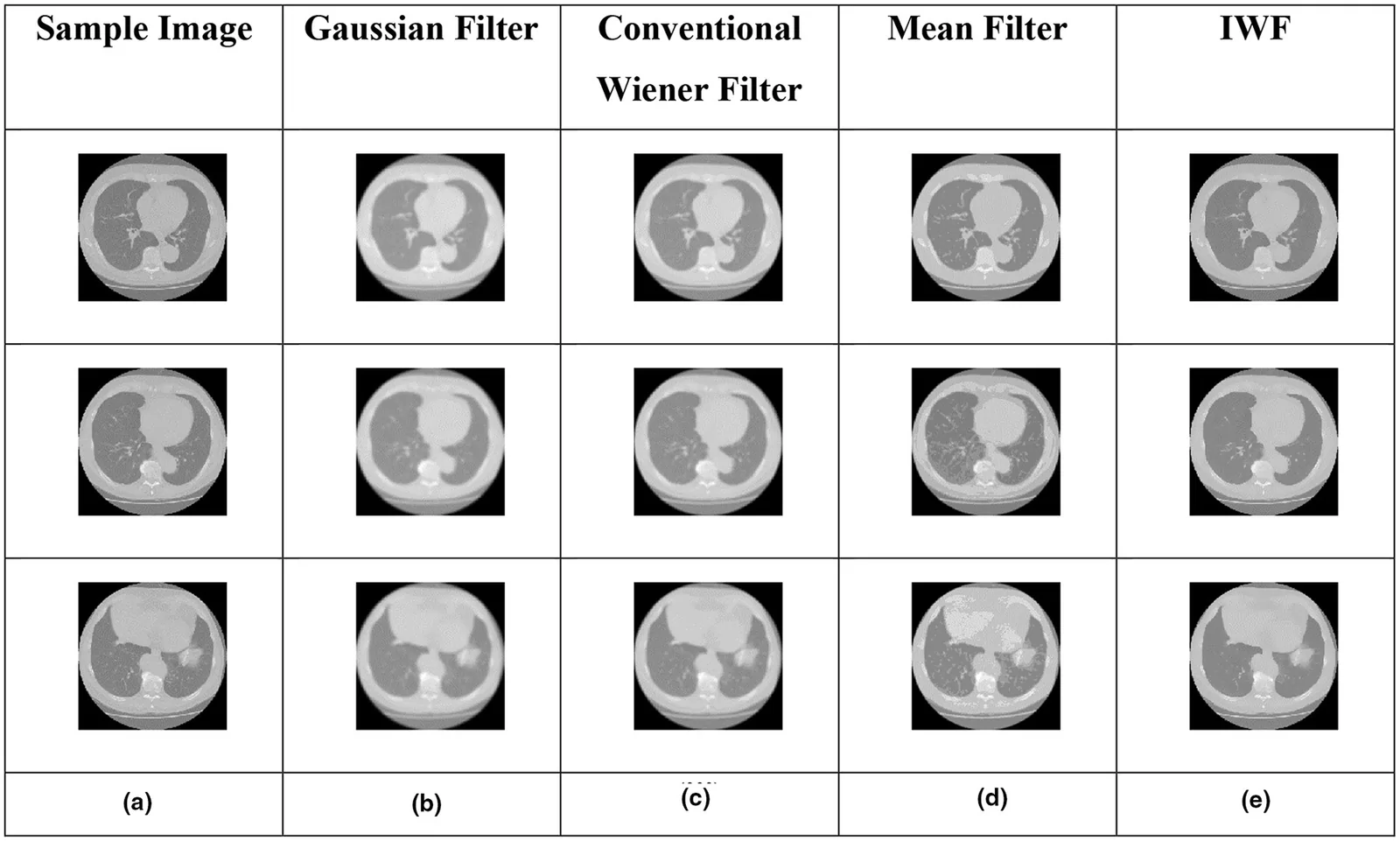
Filtered image outcomes of the proposed **(a)** sample image; **(b)** Gaussian filter; **(c)** Conventional Wiener filter; **(d)** Mean filter; **(e)** IWF.

### 4.9 Segmentation similarity metrics

Within this section, the pre-processed images are further subjected to the segmentation stages, and their reliability is measured using the Jaccard and Dice metrics. Regarding these metrics, we compute how accurately the models segment the tumor regions. The Jaccard index states the amount of correspondence between the predicted and actual tumor areas, and the Dice coefficient presents the intersection between the predicted and actual tumor areas as defined in Equations 24, 25.


$$\begin{array}{c} Dice=\frac{2|L⋂K|}{\left|L|+|K\right|} \end{array}$$ (24)

$$\begin{array}{c} Jaccard=\frac{\left|L\cap K\right|}{\left|L\cup K\right|} \end{array}$$ (25)

The segmentation process is performed by different baseline and proposed methods, and their predicted results are tabulated in Table 9. The observation shows that the Fuzzy C-Means (FCM) segmentation method achieved an extremely low segmentation accuracy of 80.7%, with Dice and Jaccard scores of 0.753 and 0.738. When comparing the proposed TransUNET and the conventional TransUNET, the MTransUNET method achieved the highest segmentation accuracy of 97.8%, with higher metric values (Dice of 0.966 & Jaccard of 0.954). This obtained the maximal ranges by enhancing residual connections and GeLU activation in the transformer.

**Table 9:** Jaccard and Dice validation of the proposed approach.

Methods	FCM	UNET	ResUNET	Conventional TransUNET	MTransUNET
Dice	0.753	0.915	0.876	0.945	0.966
Jaccard	0.738	0.892	0.888	0.935	0.954
Segmentation accuracy	0.807	0.927	0.897	0.946	0.978

#### 4.9.1 Segmentation outcome

The segmentation results for MTransUNET and the conventional TransUNET are shown in Figure 12.

**Figure 12:**
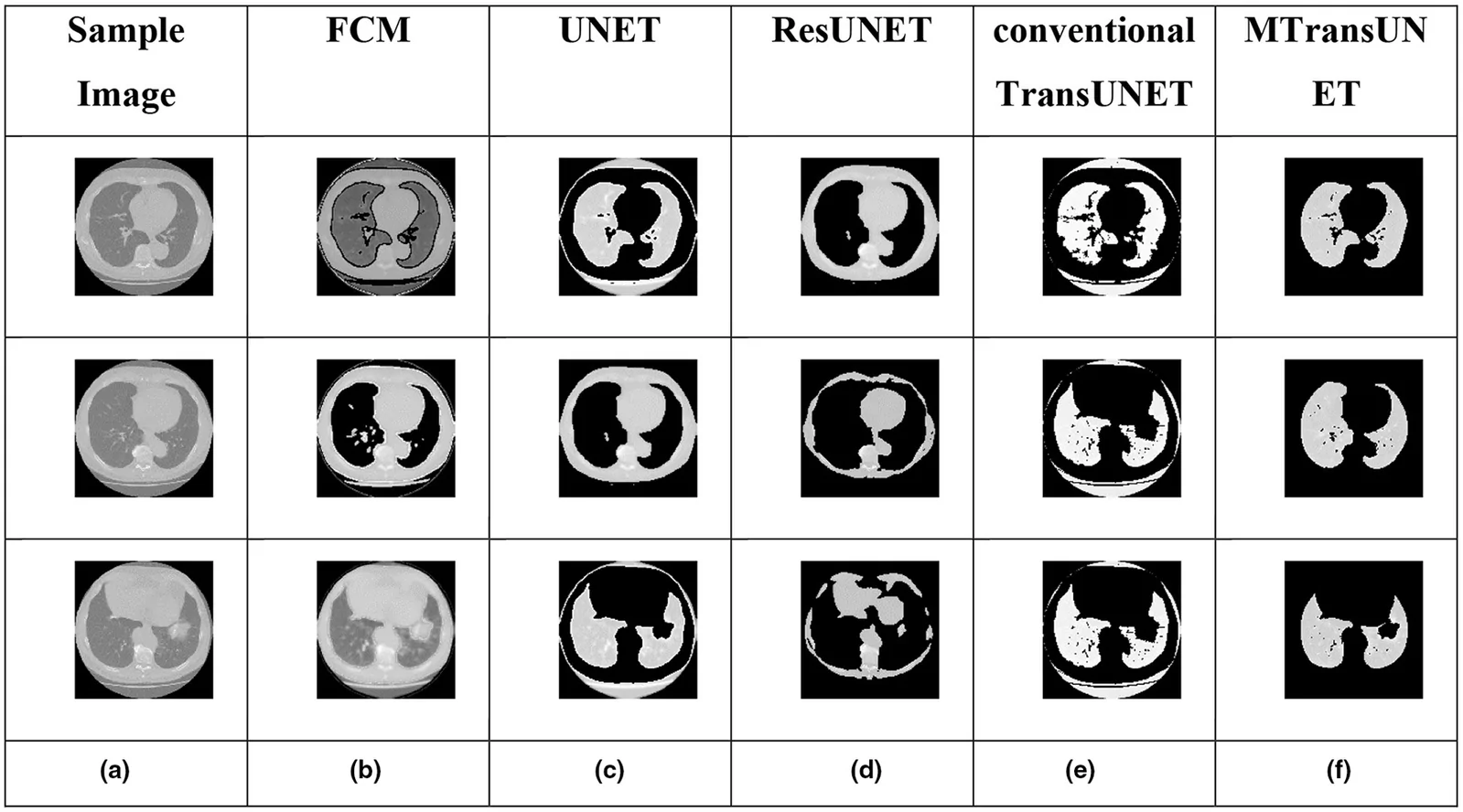
Filtered image outcomes of the proposed **(a)** sample image; **(b)** FCM; **(c)** UNET; **(d)** ResUNET; **(e)** conventional TransUNET; **(f)** MTransUNET.

### 4.10 Assessing lung cancer classification with ROC-AUC

Under this concept, the Receiver Operating Characteristic–Area Under the Curve (ROC-AUC) curve distinguishes the cancerous and non-cancerous cases by plotting the true positive rate (TPR) and FPR ranges over the different threshold points. The AUC summarizes the overall ability of models to classify correctly. An AUC closer to 1 means the model effectively identifies the cancer cases while keeping false positives low, whereas an AUC near 0.5 indicates that the model struggles to differentiate the cancerous and healthy cases. The final curve statement says that the baseline parallel CNN and EfficientNet models have raised the minimal AUC area to 95 and 93%. The IPCNN-ViT approach has enhanced the classification framework to result in the maximal AUC score of 98.5% in Figure 13.

**Figure 13:**
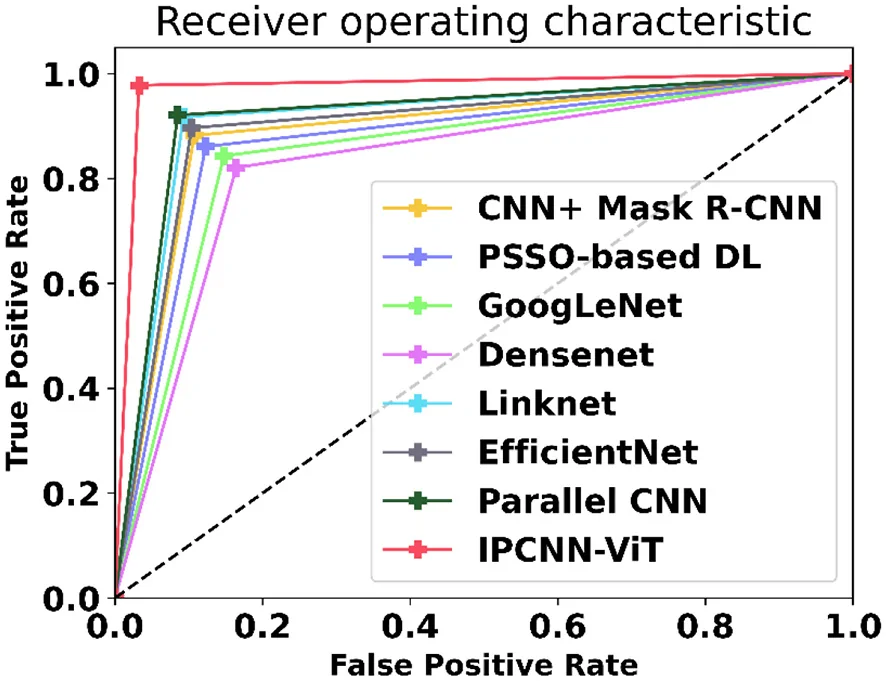
ROC-AUC estimation of IPCNN-ViT classifier.

### 4.11 Cross-dataset validation analysis

Cross-dataset validation was conducted to examine the generalization and robustness of the proposed model across different datasets. In Table 10, Dataset 1 is used for training and Dataset 2 for testing, and then the process is reversed (Dataset 2 for training and Dataset 1 for testing). When Dataset 1 is trained and Dataset 2 is tested, the algorithm yields an accuracy rate of 96.9%, a sensitivity of 97.4%, a specificity of 96.4%, a precision of 96.5%, an F-measure of 96.9%, an MCC value of 93.8%, and an NPV of 97.4% while attaining extremely low rates of FPR and FNR of 3.6 and 2.6%, respectively. When Dataset 2 is used for training and Dataset 1 for testing, the corresponding accuracy, sensitivity, specificity, precision, F-measure, MCC, and NPV are 95.9, 96.0, 95.7, 95.8, 95.9, 91.7, and 95.9%, respectively, with FPR and FNR of only 4.3 and 4.0%, respectively. These results are more accurate, robust, reliable, and capable of generalizing effectively.

**Table 10:** Analysis of cross dataset validation.

Measures	Training with dataset 1 and testing with dataset 2	Training with dataset 2 and testing with dataset 1
Accuracy	0.969	0.959
Sensitivity	0.974	0.960
Specificity	0.964	0.957
Precision	0.965	0.958
F_measure	0.969	0.959
MCC	0.938	0.917
NPV	0.974	0.959
FPR	0.036	0.043
FNR	0.026	0.040

### 4.12 Computational complexity analysis

Training time is the amount of time taken by the model to learn the patterns and parameters from the training dataset. Inference time refers to the time taken by the trained model to make predictions on an unseen input image. Table 11 presents the computational complexity analysis. The proposed method achieves a training time of 64.000 s and an inference time of 0.0299 s, demonstrating efficient computational performance. Among the methods compared, DenseNet has the lowest training time (63.849 s) and inference time (0.0298 s), whereas the PSSO-based DL has the highest training time (91.217 s) and inference time (0.0426 s). Similarly, the inference time of 0.0299 s is relatively low compared to other models and almost similar to that of DenseNet. Thus, the results provide a favorable balance between classification performance and computational efficiency is more suitable for rapid lung cancer classification and severity estimation.

**Table 11:** Analysis on training and inference time.

Methods	Training time (s)	Inference time (s)
CNN + Mask R-CNN	87.266	0.040
PSSO-based DL	91.217	0.0426
GoogLeNet	85.944	0.0401
DenseNet	63.849	0.0298
Linknet	69.069	0.0322
EfficientNet	76.847	0.0359
Parallel CNN	71.956	0.0336
IPCNN-ViT	64.000	0.0299

## 5 Conclusion

Early detection of lung cancer is crucial to provide improved prognosis and to reduce deaths worldwide. Mostly, CT scans are utilized for lung cancer classification, but that is challenging because diverse features characterize this disease. Consequently, we have introduced LCCSE-IPCNN-ViT, which is a lung cancer classification and severity estimation model that includes three phases. First, using IWF, the input image was pre-processed. Subsequently, segmentation using MTransUNet was performed. Finally, lung cancer classification and severity estimation were performed using a hybrid model named IPCNN-ViT. The experimental outcomes demonstrated this LCCSE-IPCNN-ViT model's accuracy and sensitivity. The IPCNN-ViT approach has markedly outperformed the baseline mislabeling activity in lung cancer classification, achieving the lowest FNE and FPR of 1.33 and 1.04%, respectively, at 90%. There are several limitations to the proposed LCCSE-IPCNN-ViT model. The first limitation is that the LCCSE-IPCNN-ViT model was tested on the LIDC-IDRI dataset; therefore, variability in CT scanners, imaging methods, image quality, and patient populations may affect the model's ability to generalize to other clinical environments. Another limitation of the model is that its performance depends on the accuracy of the data labels for segmentation, classification, and severity. Additionally, the four severity-labeling categories require further validation against clinical guidelines. The evaluation is also retrospective and would require further validation based on other independent, multicenter, and prospective clinical datasets.

Further studies would aim to validate the suggested framework using larger, multi-center, and more diverse CT datasets to ensure its generalizability across varying imaging conditions. The proposed framework can be further developed by incorporating clinical and multimodal data to better classify cancer and assess its severity. In addition, other studies could be carried out focusing on the use of interpretable AI methods.

## Data Availability

The original contributions presented in the study are included in the article/supplementary material, further inquiries can be directed to the corresponding author.
